# Antimicrobial Transformation Products in the Aquatic Environment: Global Occurrence, Ecotoxicological Risks, and Potential of Antibiotic Resistance

**DOI:** 10.1021/acs.est.2c09854

**Published:** 2023-06-19

**Authors:** Paul Löffler, Beate I. Escher, Christine Baduel, Marko P. Virta, Foon Yin Lai

**Affiliations:** † Department of Aquatic Sciences and Assessment, Swedish University of Agricultural Sciences (SLU), Uppsala SE-75007, Sweden; ‡ Department of Cell Toxicology, Helmholtz Centre for Environmental Research, UZ, 04318 Leipzig, Germany; § Eberhard Karls University Tübingen, Environmental Toxicology, Department of Geosciences, 72076 Tübingen, Germany; ∥ Université Grenoble Alpes, IRD, CNRS, Grenoble INP, IGE, 38 050 Grenoble, France; ⊥ Department of Microbiology, Faculty of Agriculture and Forestry, University of Helsinki, 00014 Helsinki, Finland; # Multidisciplinary Center of Excellence in Antimicrobial Resistance Research, Helsinki 00100, Finland

**Keywords:** metabolites, surface water, micropollutants, environmental analysis, degradation products, antimicrobial resistance, risk assessment, chemical prioritization

## Abstract

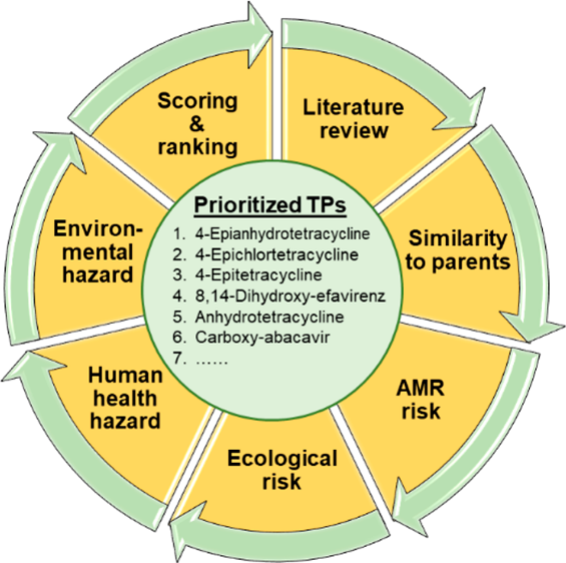

The global spread of antimicrobial resistance (AMR) is concerning for the health of humans, animals, and the environment in a One Health perspective. Assessments of AMR and associated environmental hazards mostly focus on antimicrobial parent compounds, while largely overlooking their transformation products (TPs). This review lists antimicrobial TPs identified in surface water environments and examines their potential for AMR promotion, ecological risk, as well as human health and environmental hazards using *in silico* models. Our review also summarizes the key transformation compartments of TPs, related pathways for TPs reaching surface waters and methodologies for studying the fate of TPs. The 56 antimicrobial TPs covered by the review were prioritized via scoring and ranking of various risk and hazard parameters. Most data on occurrences to date have been reported in Europe, while little is known about antibiotic TPs in Africa, Central and South America, Asia, and Oceania. Occurrence data on antiviral TPs and other antibacterial TPs are even scarcer. We propose evaluation of structural similarity between parent compounds and TPs for TP risk assessment. We predicted a risk of AMR for 13 TPs, especially TPs of tetracyclines and macrolides. We estimated the ecotoxicological effect concentrations of TPs from the experimental effect data of the parent chemical for bacteria, algae and water fleas, scaled by potency differences predicted by quantitative structure–activity relationships (QSARs) for baseline toxicity and a scaling factor for structural similarity. Inclusion of TPs in mixtures with their parent increased the ecological risk quotient over the threshold of one for 7 of the 24 antimicrobials included in this analysis, while only one parent had a risk quotient above one. Thirteen TPs, from which 6 were macrolide TPs, posed a risk to at least one of the three tested species. There were 12/21 TPs identified that are likely to exhibit a similar or higher level of mutagenicity/carcinogenicity, respectively, than their parent compound, with tetracycline TPs often showing increased mutagenicity. Most TPs with increased carcinogenicity belonged to sulfonamides. Most of the TPs were predicted to be mobile but not bioaccumulative, and 14 were predicted to be persistent. The six highest-priority TPs originated from the tetracycline antibiotic family and antivirals. This review, and in particular our ranking of antimicrobial TPs of concern, can support authorities in planning related intervention strategies and source mitigation of antimicrobials toward a sustainable future.

## 1 Introduction

Antimicrobial resistance (AMR) is a global health issue that affects humans, animals, and the environment. Within the One Health concept, the environment is regarded as an important compartment for the evolution and dissemination of AMR. While it occurs naturally, AMR is promoted by the widespread use of antimicrobial chemicals, such as antibiotics, which can induce bacterial resistance and lead to loss of antimicrobial function in treating infections. Almost five million global deaths in 2019 were estimated to be associated with AMR.^1^ In 2019, the World Health Organization (WHO) named AMR as one of the top 10 threats to global health and called for a reduction in the spread of AMR from all potential sources.^2^ Nevertheless, studies show increasing global demand and usage of antimicrobial chemicals in both humans^3^ and animals.^4^ For example, global per-capita antibiotic consumption increased by 39% in the period 2000–2015.^5,6^ Apart from a few high-income countries (e.g., Hong Kong, Japan, Singapore, Hungary, France, and United States), most countries have increased their antibiotic consumption, with low- to middle-income countries in particular having increased their consumption by up to 30 daily defined doses per 1000 inhabitants.^5,6^

Since the discovery of the most prominent, penicillin,^7^ a broad range of antibiotics have been developed and assigned to various classes, e.g., β-lactams, tetracyclines, macrolides, sulfonamides, and quinolones. Target-specific antivirals are another group of antimicrobials assigned to a number of classes.^8^ Antimicrobial medications are excreted from the treated subject in unchanged form (parent compounds) or metabolized to other chemical forms (metabolites or biotransformation products (bioTPs)), which collectively end up at wastewater treatment plants (WWTPs). Studies have shown that conventional WWTPs are inefficient in removing the wide variety of antimicrobial residues in wastewater and may convert them into other chemical forms (treatment TPs). Remaining residues and treatment TPs are released together to the aquatic environment via effluent discharge.^9,10^ Conventional biological treatments, additional treatment steps such as removal by adsorption^11−13^ and filtration,^14,15^ and several more advanced treatment techniques (e.g., advanced oxidation,^16−18^ reverse osmosis,^19−21^ or electrochemical degradation^22−24^) have been investigated, but complete removal of the entire suite of antimicrobial classes by one single treatment method remains challenging.^25−27^ Owing to inefficient treatment methods and the fact that 44% of domestic wastewater is still not safely treated globally,^28^ many studies have reported the presence of antimicrobial parent compounds and related TPs in aquatic environments worldwide.^29−35^ As the COVID-19 pandemic only slowly recedes,^36^ the occurrence of antivirals used for treating COVID-19 and their TPs in water is also expected.^37^

Besides use in humans, antimicrobial chemicals are also used extensively in animal husbandry, plant production, and aquaculture, to ensure animal health and a safe food supply.^38^ Wastewater and runoff water from these sectors (e.g., from livestock wastewater treatment plants) and from manure-treated farmland can act as diffuse sources of antimicrobial chemicals and their TPs in aquatic environments.^38,39^ Use of higher volumes of antibiotics for animals than for humans has been reported for 8 of 29 European countries,^40,41^ but the average (biomass-corrected) consumption rate of antimicrobials is similar for humans and food-producing animals.^41^

TPs often have similar molecular structure to their parent chemical and may thus show similar environmental behavior and biological activity. Previous studies have suggested that some TPs may pose a similar or greater risk to aquatic environments than their active parent compound.^42,43^ However, compared with the parent antimicrobial compounds,^44−46^ little is known about the aquatic occurrence of their TPs and resulting ecological effects and promotion of AMR. The key aims of this review were to provide an overview of the global occurrence of antimicrobial TPs in aquatic environments and to prioritize TPs based on structural similarity between the TPs and their corresponding parent compounds and also potential hazards of the TPs. The risk was assessed considering four aspects: risk quotient for promotion of AMR (RQ_AMR_); ecological risk (RQ_species_); mutagenicity and carcinogenicity, as proxies for human health hazard; and persistence, mobility, and bioconcentration potential, as environmental hazard indicators. These indicators were scored against criteria and ranked to prioritize a list of antimicrobial TPs of highest concern.

## 2 Meta-analysis

### 2.1 Data Compilation

**Figure 1:**
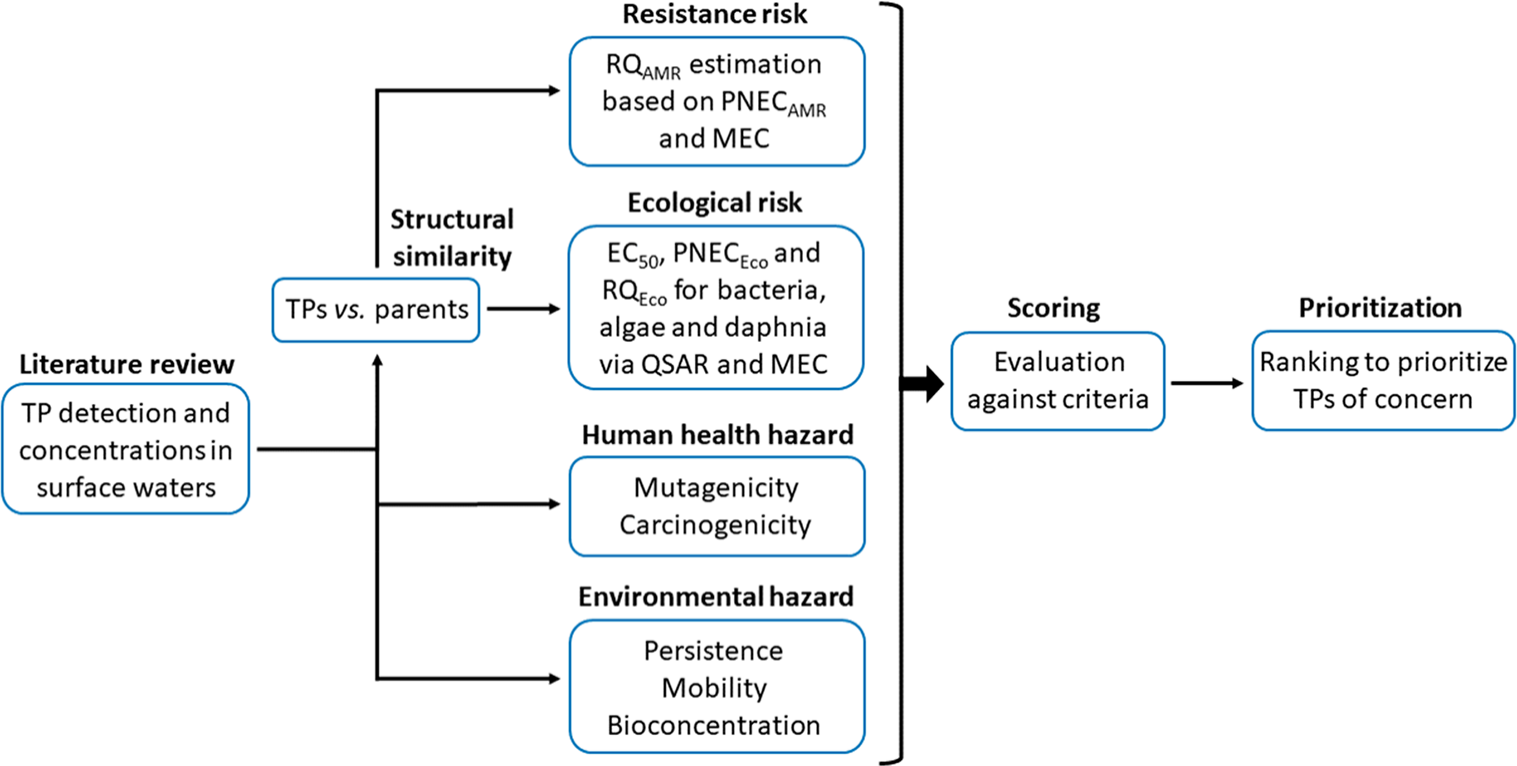
Workflow of meta-analysis in this review to prioritize TPs of concern in surface water environments. RQ_AMR_: risk quotient of antimicrobial resistance; RQ_eco_: ecological risk quotient concerning three different species (RQ_species_); PNEC_AMR_: predicted no-effect concentration for antimicrobial resistance; PNEC_eco_: predicted no-effect concentration for ecological risk; MEC: measured environmental concentrations; EC_50_: 50% effect concentration.

We searched the Web of Science and PubMed literature databases, using search terms including “antimicrobial or antibiotic” and “metabolite or transformation product” and “surface water”, to locate relevant publications in English available by September 30, 2021. In this review, the term antimicrobial refers to antibiotic, antibacterial, and antiviral compounds. A total of 7247 articles were initially obtained (Figure S1). Duplicates in the databases were removed and articles were screened considering antimicrobial TPs in surface waters. Based on these findings and supplementing with cross-references, we finally selected 75 research articles (Table S1). The meta-analysis (Figure 1) of the compiled data was performed as described in the following sections.

### 2.2 Similarity Evaluation

The structural similarity of a TP to its parent compound was assessed using two different similarity measures. As a 3D similarity measure, we used the Augmented Lagrangian algorithm in the MolShaCS software,^47^ in which the underlying Gaussian base function separates the charge distribution into positive and negative parts and calculates similarity as Hodgkin’s index.^48,49^ As a 2D similarity measure, we used the 2D-similarity workbench of the ChemMine Tool,^50^ which assesses similarity via maximum common substructure (MCS) with Tanimoto coefficient.^51^ We considered TPs with similarity >0.998 using MoklShaCS (3D) or >0.95 using MCS (2D) as having high similarity, and TPs with lower similarity values as showing low/no similarity. These values agree with the known activity loss of β-lactam TPs via ring-opening.

### 2.3 Resistance Risk Assessment

Since few data are available on the antimicrobial activity of the identified TPs, we used predicted no-effect concentrations for resistance selection (PNEC_AMR_) of the respective parent compound^52^ as the threshold in calculating the risk quotient of resistance selection (RQ_AMR_). PNEC_AMR_ was estimated based on minimal selective concentrations using minimum inhibitory concentrations (MICs).^52^ For TPs meeting the similarity criterion, we divided their highest measured environmental concentration (MEC, Tables S1 and S2) by the PNEC_AMR_ of the parent compound (eq 1). Most of the TPs have quantitative data as target analysis was applied. For 12 TPs detected via suspect and nontargeted approaches, 1 ng L^–1^ as the potential lowest limit of high-resolution mass spectrometry was assigned for the RQ calculations. For TPs classified as dissimilar, a lower effect potency was assumed, and a factor of 10 was applied to estimate RQ_AMR_ (eq 2).
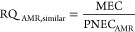
1
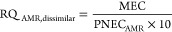
2

### 2.4 Ecological Risk Assessment

We compiled available experimental ecotoxicity data for parent compounds and TPs from primary literature. Since very few experimental effect data on TPs were found, we predicted the ecotoxicological hazard of the TPs using a combination of baseline toxicity and specificity of the respective parent compound. We reformatted quantitative structure–activity relationships (QSARs) for baseline toxicity based on log *K*_ow_ for ionizable organic chemicals using the ionization-corrected liposome–water distribution ratio at pH 7 (*D*_lipw_) as a hydrophobicity descriptor according to the studies of Escher et al.^53,54^ (eqs 3–5). Although the applicability domain varied in the original log *K*_ow_-based QSARs, we predicted the baseline toxicity for all chemicals and TPs with log *D*_lipw_ > 0.

We retrieved data on physicochemical properties (octanol–water partitioning coefficient *K*_ow_, acidity constant p*K*_a_) from the U.S. EPA’s Estimation Programs Interface EpiSuite.^55^ Since most TPs had no experimental data available, we predicted *K*_ow_ with OPERA,^56^ using the CompTox Chemicals Dashboard,^57^ and p*K*_a_ with ACD/p*K*_a_.^58^ We estimated *D*_lipw_ from the speciation (fraction of species *i*, α_*i*_) and the liposome–water partition constant *K*_lipw_ (eq 6).^54^ We derived the log *K*_lipw_ of the neutral species from log *K*_ow_ using eq 7 and used one log unit lower for all charged species.^54^

*Aliivibrio fischeri* (formerly named *Vibrio fischeri*)^59^
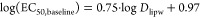
3*Pseudokirchneriella subcapitata*(53)
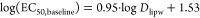
4*Daphnia magna*(60)
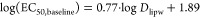
5Liposome–water distribution ratio at pH 7^54^
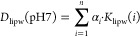
6Liposome–water partitioning constant^54^
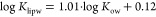
7

Baseline toxicity is the minimal toxicity. If a chemical has a specific mode of toxic action, it has higher toxicity and lower EC_50_, which can be quantified by the toxic ratio (TR, eq 8). At TR > 10, a chemical can be considered to act specifically.^61^ Due to experimental uncertainty, often caused by solubility issues or other experimental challenges, the TR derived from EC_50,experimental_ can sometimes have values <1. For TPs with high similarity, we considered the TR of TPs to be equivalent to that of parent compounds (TR(P), eq 8) and thus applied the TR of the respective parent compound to the estimated baseline EC_50_ (pH 7) to obtain an EC_50,specific_(similar TP) estimate (eq 9). For TR(P) < 1, TR(TP) was set to 1, the minimum theoretical TR. For TPs with low similarity, we divided the TR of the parent compounds by 10 before estimating EC_50,specific_(dissimilar TP) (eq 10). If the TR(P)/10 was <1, we adjusted it to 1, because no chemical can have lower effects than baseline toxicity, unless it is unstable or metabolized.
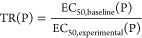
8
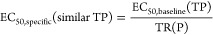
9
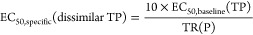
10

We estimated the predicted no-effect concentration (PNEC_eco_) for aquatic ecosystems through dividing the EC_50_(TP) by the assessment factor for freshwater organisms, according to the European Chemicals Regulation REACH.^62^ Since the selected baseline toxicity QSARs referred to acute toxicity, we applied an assessment factor of 1000 (eq 11). Strictly speaking the minimum EC_50_ of the three species EC_50_ would have to be used to derive the PNEC_eco_ protective for the ecosystem, but for illustration purposes we derived PNECs for each species (PNEC_species_) individually.
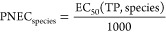
11

We calculated ecological species risk quotient (RQ_species_) through dividing the highest determined MECs (Tables S1 and S3) by the PNEC_species_ (eq 12). Same as for the resistance risk assessment, quantitative MECs were used for most of the TPs, while an MEC of 1 ng L^−1^ was assigned for 12 TPs detected via suspect and nontarget approaches in the RQ calculation.
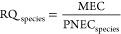
12

The parent compounds’ surface water concentrations compiled from this literature review were used for an estimate of the parent risk. The mixture risk of parent compound occurring with the respective TP was assessed using the concentration addition approach, which sums up the risk quotient of the parent and all TPs (eq 13).^63^
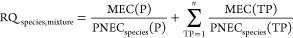
13

The RQ for the entire ecosystem is then defined (eq 14) in relation to the PNEC_eco_ = min(PNEC_species_).
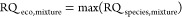
14

### 2.5 Human Health Hazard

We assessed the potential of mutagenicity using CONSENSUS v.1.0.3, and carcinogenicity using the CAESAR v.2.1.9 model in VEGA QSAR (v.1.1.4).^64^ We validated the predictions using 26 randomly chosen compounds from the EURL ECVAM Genotoxicity and Carcinogenicity Consolidated Database of Ames Positive Chemicals (Table S4)^65^ and the predictions for the parent compounds by comparing them to experimental literature (Table S5). The model performance was evaluated based on sensitivity, selectivity, accuracy, and Matthews correlation coefficient (MCC, to counter skewed data), in accordance with Benfenati et al.^66^ (Table S6). We evaluated model outputs according to their specified reliability and consensus score (only applicable for mutagenicity), where experimental values were considered the highest level of certainty. We also evaluated unchanged or changed (increase or decrease) predicted mutagenicity or carcinogenicity for TPs relative to the respective parent compound (Table S7−S8).

### 2.6 Environmental Hazard Predictions

As supplementary descriptive factors, we estimated environmental hazard indicators for TPs, including persistence, bioconcentration factor (BCF), and mobility, using the VEGA software. It should be noted that these estimates are tentative and have to be treated with caution, since ionizable organic compounds may not always fall within the applicability domain of the prediction models,^67^ and also none of the models used antimicrobial parent compounds or TPs compiled in this review in their training and validation data sets. We predicted TP persistence (half-life in water, in days) with the quantitative model IRFMN v.1.0.0^64^ and bioaccumulative properties with the BCF model Meylan v.1.0.3. We identified mobile compounds based on water solubility estimated using the IRFMN model v.1.0.0 and estimated *K*_OC_ using the OPERA v.1.0.0 model.

### 2.7 Scoring and Prioritization

In the last step of our meta-analysis (Figure 1), we assigned TPs a score between 0 and 1 for each parameter in relation to the criteria (Table 1). We assigned a score of 0 (risk) for RQ_AMR_ and RQ_species_ values higher than 1 and a score of 1 (no risk) below 1. For 24 TPs, the PNEC_AMR_ of the respective parent compound was unavailable, and thus their RQ_AMR_ could not be calculated and were conservatively assigned a score of 0. Similarly, 23 compounds were outside the applicability domain of the QSAR and were conservatively classified to pose a risk. For mutagenicity and carcinogenicity, we assigned a hazard (score 0) when the TP showed similar mutagenicity/carcinogenicity to the parent compound or when an increase in the predicted mutagenic/carcinogenic probability was observed. We assessed the criteria for persistence, mobility, and BCF in accordance with the REACH regulation guidelines.^68,69^ We assigned a score of 0 for estimated persistence greater than 40 days, solubility greater than 150 μg L^–1^ or log *K*_OC_ ≤ 4.5, and BCF^70^ greater than 3.3; otherwise, a score of 1 was assigned. We then added the scores together and ranked the TPs from low to high scores, reflecting TPs of high to low concern, respectively (Table S8).

**Table 1:** Parameters and Related Criteria in the Scoring System for Prioritization of TPs^a^

**Parameter**	**Score 0**	**Score 1**
AMR risk	RQ_AMR_ > 1	RQ_AMR_ < 1
Ecological risk	RQ_species_ > 1	RQ_species_ < 1
MC (mutagenicity or carcinogenicity)	MC_TP_ > MC_P_ (TP shows MC)	MC_TP_ < MC_P_ (TP does not show MC)
Persistence^68^	>40 days	<40 days
BCF^68^	log BCF > 3.3	log BCF < 3.3
Mobility^69^	Solubility > 0.15 mg L^–1^ and log *K*_OC_ ≤ 4.5	Solubility < 0.15 mg L^–1^ and log *K*_OC_ ≥ 4.5

a AMR = antimicrobial resistance; RQ = risk quotient; BCF = bioconcentration factor.

## 3 Key Sources and Transformation Pathways

**Figure 2:**
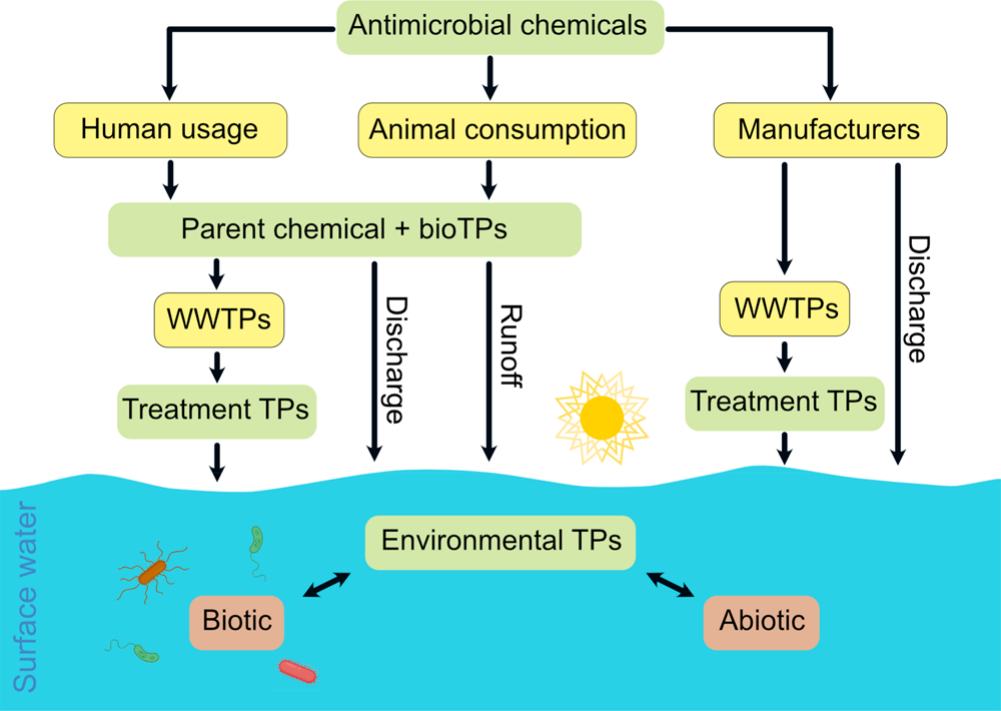
Major sources, pathways and processes of converting antimicrobial chemicals into their TPs in different environmental compartments. WWTPs: wastewater treatment plants where (a)biotic transformation processes can occur; bioTPs: TPs formed by human and animal metabolism.

Aquatic environments receive antimicrobial residues, i.e., parent compounds and different kinds of TPs, from various sources, including untreated wastewater, effluent discharge from WWTPs and pharmaceutical factories, and runoff from aquaculture and animal husbandry (Figure 2). After ingestion, antimicrobial chemicals are biotransformed in humans and animals via phase I and/or phase II metabolism, resulting in the formation of bioTPs.^71−74^ Different reactions are described in the literature (Tables S9–S11), such as conversion of metronidazole to hydroxymetronidazole (phase I bioTP) via oxidation (Table S10),^75,76^ or sulfamethoxazole (SMX) to SMX-*N*-glucuronide (phase II bioTP) via reduction followed by conjugation with glycosides (Table S10).^76−78^ The relative proportion of bioTPs to parent compound excreted varies between antimicrobial chemicals and organisms.^80,81^ Further, (a)biotic transformations of the excreted parent compounds and/or bioTPs may occur at WWTPs depending on the treatment steps implemented,^82^ resulting in generation of treatment TPs. Due to inefficient treatment techniques, excreted parent compounds and/or bioTPs are often detected in effluent wastewater from municipalities and hospitals.^34,35^

In aquatic environments, photochemical reactions induced by natural light are reported to be one of the major degradation/transformation pathways for antimicrobial chemicals,^83^ resulting in production of abiotic environmental TPs. In direct photolysis, irradiation with ultraviolet (UV) and visible light (290–800 nm) allows energy transfer from photon to electrons in a molecule, which is then promoted to an unstable, energetically excited state that leads to bond cleavages or further chemical reactions and molecular rearrangements. In indirect photolysis, natural compounds occurring in the aquatic environment (namely, photosensitizers) can absorb light and produce reactive oxygen species, which subsequently react and transform the antimicrobial chemicals.^83^ These (in)direct reactions are strongly influenced by light availability and water quality in the aquatic system (depth, turbidity, chemical composition, etc.) and by irradiation intensity (depending on season, weather, altitude, and latitude).

It should be noted that, since (bio)transformations can occur in different compartments (Figure 2), we found it challenging to classify a TP specifically as a bioTP or (a)biotic environmental TP. For example, anhydroerythromycin was reported as various types of TP in 18 of the articles reviewed (Table S10), and methyl triclosan was reported as various types of TP in 8 articles (Table S11). Some articles provided no further indication of the processes resulting in the studied (environmental) TPs, e.g., carboxy-acyclovir or emtricitabine *S*-oxide (Table S9).

## 4 Approaches to Generating and Identifying TPs

### 4.1 Laboratory Experiments

#### 4.1.1 Abiotic Photolytic Transformation

Many researchers have conducted laboratory scale experiments to study (in)direct phototransformation of antimicrobial chemicals.^83−89^ Those studies have revealed that the structure and/or abundance of photo-TPs generated is highly affected by light quality (wavelength and intensity) and by water composition. Based on the respective bond energies, photochemical reactions are most common between 260 and 820 nm.^91^ In microcosm experiments, phototransformation has been studied using different light sources, including UV light, solar simulation,^84,85,90,92,93^ and natural light.^93−96^ OECD guideline no. 316 for the phototransformation of chemicals in water recommends a xenon lamp with a wavelength range of 290–800 nm.^97^ The spectral power distribution of the artificial light source is crucial for extrapolation of the results to environmental conditions. Misconceptions can arise from the use of different instruments that set the spectral power distribution over a different range of wavelengths (e.g., 300–400 nm or 300–800 nm). Moreover, despite applying the same range of wavelengths, the intensity may differ.^85,98,99^ Even when the spectrum applied to the sample is the same, the difficulty in conversion of irradiation adjusted using different spectral ranges (e.g., 300–400 nm or 300–800 nm) could result in perceived differences, e.g., 60.5 W m^–2^ adjusted via the UV range (300–400 nm) is the same intensity as 550 W m^–2^ adjusted via the UV–visible range (300–800 nm).^100^ Therefore, we recommend that future studies report the applied wavelength, spectral power distribution, and adjustment range of the wavelength, for clearer interpretation of the results. Furthermore, the addition of chromophore compounds, which undergo a light-induced reaction for which the quantum yield is known accurately (chemical actinometer), could be beneficial to calibrate the light intensity.^101^

The effect of environmental conditions on photolysis can be studied in microcosm experiments using various water matrices, such as fresh water and seawater.^85,98,99,102^ The role played by many chemical constituents, such as dissolved organic matter and inorganic ions (e.g., Cl^–^, NO_3_^–^, and CO_3_^2–^), in phototransformation kinetics and photolytic pathways can be investigated in this way. The main photosensitizers occurring in surface waters are nitrate and dissolved organic matter. Irradiation of nitrate and organic matter can produce light-excited organic matter and oxidant species, e.g., hydroxyl radicals (·OH), singlet oxygen (^1^O_2_), or superoxide anion (O_2_^–•^).^83^ All of these factors influence the formation of TPs. Consequently, antimicrobial chemicals can have various abiotic environmental TPs in surface water (Tables S9–S11). For example, amoxicillin (AMX) has been reported to have four different abiotic environmental TPs,^101−104^ namely, 3-(4-hydroxyphenyl)pyrazinol, AMX penilloic acid, AMX penicilloic acid, and AMX 2′5′-diketopiperazine (Table S10). High concentrations of photosensitizers in surface water can also hinder antimicrobial photolysis.^107^ Some studies report an influence of pH on light absorbance for transforming antimicrobial chemicals. The pH of surface waters is typically between 4 and 9,^108,109^ which is within the p*K*_a_ range of some antimicrobial chemicals and can thus affect their ability to absorb light.^110^ For example, Jin et al.^111^ identified pH as the key factor controlling the direct photolysis rate of oxytetracycline. Those authors observed an increased photolysis rate of oxytetracycline with increased pH, which was associated with inter-/intramolecular proton transfers.

As the types and abundances of photo-TPs are highly dependent on the phototransformation pathway, it is necessary to study as many phototransformation pathways as possible to comprehensively identify the TPs of antimicrobial chemicals. Most studies on antimicrobial chemicals focus on direct photolysis, while, for example, reactions with OH–radicals and indirect photolysis induced by dissolved organic matter are often less well investigated.^108−110,112-114^

#### 4.1.2 Biotic Transformation

To investigate environmental biotic transformation, surface water with an intact microbiome is needed. Hence, laboratory microcosm experiments are best performed as soon as the water is sampled. If this is not feasible, storage for up to 4 weeks at 4 °C can be tolerated.^115^ According to OECD guideline no. 309, water should be taken from sites where no known contamination with the substance of interest has occurred in the past. To investigate biodegradation rates, environmentally relevant concentrations of the chemical of interest should be used in microcosm experiments. Cultures without prior contact to the antimicrobials of interest help avoid any distortion of the bacterial community. For identification of bioTPs, a high concentration of the parent compound could be used to generate sufficient amounts of TPs, avoiding analytical limitations.^115^

Another approach to studying environmental biotransformation is to use ^14^C-radiolabeled chemicals. Girardi et al.^116^ used radiolabeled ciprofloxacin to investigate biodegradation of this compound in surface waters based on its CO_2_ evolution, following OECD guideline no. 301B.^117^ Although several antimicrobials are known to be unaffected by most common wastewater treatment processes, only a few studies have investigated biotransformation processes in surface waters and the interaction with water and sediment.^118,119^ The biodegradation rate has been shown to be dependent on water type and its microbiome, e.g., Baena-Nogueras et al.^99^ observed that biodegradation was enhanced by a seawater microbiome in comparison with a surface water microbiome. Patrolecco et al.^120^ investigated the effect of the copresence of ciprofloxacin on the biotic transformation of sulfamethoxazole and found no significant difference in biotic degradation rates. They also compared the biodegradation rate with photolysis and found a synergic effect of the two processes.^120^ Further, parameters that can ensure the studied systems proper function should be mentioned, such as measurements of oxygen concentrations to confirm aerobic conditions and measurements of Fe(II) to confirm anaerobic conditions. pH measurements can also give insights into the system conditions because the pH might change under aeration.^117,121^

Hydrolysis through enzyme-mediated nucleophilic reactions by hydrolases is one of the main biological transformation reactions, and it occurs under all environmental conditions.^122,123,83^ A second degradation reaction is oxidation using an electrophilic form of oxygen or bio-oxidants (e.g., mono-, dioxygenase). This reaction is generally only possible in aerobic environments.^83,124,125^ Regarding human TPs, mostly the main metabolites have been investigated, while other bioTPs have often not been fully assessed or are not known at all. The mammal enzyme family cytochrome P450 functions as a monooxygenase and is an important part of biotransformation via the oxidation of xenobiotic compounds.^126,127^ A third microbial reaction pathway is reduction involving nucleophiles, which includes the same structural moieties as abiotic reductions. In general, an electron withdrawing group polarizes a central atom and makes it amenable to nucleophilic attacks, in which the oxidation state of the central atom is reduced.^83^ Reductive dehalogenation represents a special case of biotic reduction involving enzymes (reductive dehalogenases, Rdases) that are able to eliminate certain halogens from organic molecules (e.g., TmrA, CfrA, VcrA).^128−132^ The proposed mechanism of reductive dehalogenation uses enzyme-bound Co^I^ as a low-potential electron donor for the electron transfer reactions.^129−133^ Reductive dehalogenation has mostly been observed under anaerobic conditions, but recent studies have reported microbial degradation of halogenated compounds under aerobic conditions.^134,135^

### 4.2 Analytical Measurement

The vast majority of studies included in this review applied target analysis using liquid chromatography coupled with tandem mass spectrometry (LC-MS/MS) to detect and quantify antimicrobial TPs in surface water (Table S1). Seven studies investigated methyl triclosan using gas chromatography coupled with mass spectrometry (GC-MS).^136−142^

Only 9 of 75 studies included in this review performed suspect or nontarget screening for the discovery and detection of antimicrobial TPs using mainly high-resolution accurate mass spectrometry (Table S1). Different approaches have been applied for identification and (semi)quantification of TPs in environmental samples. Suspect screening has been performed using libraries of known TPs,^e.g.^^104,106^ potential TPs via *in silico* prediction software (BioTransformer 3.0, Meteor, CTS, etc.),^144−147^ and/or through generation of TPs in controlled laboratory experiments.^103,105^ Due to the unavailability of analytical standards for TPs, semiquantification has been proposed to estimate approximate TP concentrations based on ionization efficiency or structurally similar compounds.^148,149^

Analysis of mostly unknown TPs is challenging from the analytical perspective, because the relevant signals in the “feature-forest” of a high-resolution chromatogram must be identified. Targeted approaches can miss potential peak exposures of untargeted compounds and have the predicament of choosing compounds of interest. Nevertheless, they provide a more accurate strategy for quantification of substances. An added strength is provided by combining nontarget approaches with targeted quantification methods. The different acquisition methods, such as data-(in)dependent acquisition or MS^n^ experiments, exceed the scope of this review but are adequately described in the compiled literature references.^149−153^

## 5 Occurrences of TPs in Surface Water Environments

### 5.1 Antivirals

Of the 75 articles reviewed, 19 investigated and verified the occurrence of eight antiviral TPs (carboxy-abacavir, carboxy-acyclovir, 8,14-dihydroxyefavirenz, carboxy-emtricitabine, emtricitabine *S*-oxide, carboxy-lamivudine, 12-hydroxynevirapine, and oseltamivir carboxylate) in surface waters (Table S9).^132,154−175^ Most of these derived from parent compounds approved for the treatment of at least one HIV strain or influenza virus (Table S12). For carboxy-acyclovir, its parent compound, acyclovir, is used for treatments of herpes simplex and varicella-zoster virus infections.^8^ Although the measured concentrations (150–200 ng L^–1^) of carboxy-acyclovir revealed no toxicity,^160,165^ acute bacterial toxicity has been found for the single oxidation product of carboxy-acyclovir (*N*-(4-carbamoyl-2-imino-5-oxoimidazolidin)formamido-*N*-methoxyacetic acid).^156,173^ This suggests a need for more research and full scrutiny of the combination of processes such as biotic and abiotic degradation mechanisms.

The majority of the studies investigating antiviral TPs (14 of 19) focused on oseltamivir carboxylate,^146,149,150,152,154,155,158−163,165,166^ the pharmacologically active human bioTP of oseltamivir, which was detected in concentrations up to 1500 ng L^–1^ during the 2009 influenza pandemic.^167^ Oseltamivir carboxylate is largely excreted (75%) following oseltamivir consumption^176,177^ and is reported to be poorly removed (<50%) at WWTPs.^175^ Azuma et al.^174^ found higher concentrations of oseltamivir carboxylate than of oseltamivir and were able to make predictions of the environmental concentrations based on the reported number of influenza patients. Similarly, Prasse et al.^159^ used MECs of oseltamivir carboxylate in surface water to evaluate the epidemic trend in influenza. As Japan accounted for about 70% of global oseltamivir consumption in 2004,^178^ the majority of the oseltamivir carboxylate-related studies reviewed (9 of 14) examined oseltamivir carboxylate in Japanese surface waters.^149,154,155,160−163,165,166^ Other antiviral TPs, such as the similarly administered active bioTP favipiravir-ribofuranosyl-5′-triphosphate (prodrug favipiravir), may also be important, particularly as that TP is known to pose a risk of teratogenicity and embryotoxicity.^179−182^

Five studies investigated and determined antiviral TPs other than oseltamivir carboxylate, including carboxy-abacavir, carboxy-acyclovir, carboxy-emtricitabine, carboxy-lamivudine, emtricitabine *S*-oxide, 8,14-dihydroxyefavirenz, and 12-hydroxynevirapine.^154,160,162,165,166^ The parent compounds of these are abacavir, acyclovir, efavirenz, emtricitabine, and lamivudine, which are approved for HIV treatment and intended to be consumed on a regular basis. Boulard et al.^162^ detected emtricitabine *S*-oxide at a maximum concentration of 380 ng L^–1^ in German surface waters, while Mosekiemang et al.^154^ detected 12-hydroxynevirapine and 8,14-dihydroxyefavirenz in concentrations of up to 4300 and 15 200 ng L^–1^, respectively. The nevirapine bioTP has been associated with severe liver and skin toxicity^182,183^ and thus may also have adverse effects on nontarget organisms. The carboxy-bioTPs, attributed to biological oxidation of hydroxyl moieties (e.g., carboxy-abacavir, carboxy-emtricitabine, carboxy-lamivudine), were mostly detected in the aquatic environment.^166^ So far, only two studies have focused on ribavirin, an antiviral medication approved for the treatment of hepatitis.^155,184^ Although those studies analyzed wastewater samples for the parent compound, they did not test for the presence of TPs of ribavirin. Since medical treatment with ribavirin is sporadic, rather than on a regular basis, and vaccines are available for hepatitis A and B, environmental concentrations can be expected to be low, and the analytical method used for detection must thus have high sensitivity. In general, few data are available about the occurrence of antiviral TPs in surface waters (Figure S2A), so more research is needed for a risk assessment.

### 5.2 Antibiotics

#### 5.2.1 Sulfonamides

Of 75 articles reviewed, 45^75−79,103−106,143,162,185−220^ detected 48 different antibiotic (bio)TPs in surface waters (Table S10). TPs from the parent compounds SMX^75−78,104,139,185,188,198−202,206−210,213^ were the most frequently reported (40%, 18 of 45 articles). A total of 18 TPs representing five classes of sulfonamides were reported (Table S10). *N*-Acetyl-SMX was most commonly studied.

After SMX consumption, around 45–70% (pH-dependent) of SMX is excreted via urine within 24 h, together with the inactive metabolite, comprising 43% *N*-AcSMX (phase I metabolite) and 9–15% SMX-*N*-glucuronide (phase II metabolite).^222,223^ SMX is widely used in veterinary prophylaxis and treatment of infections.^224^ It is also recommended for the treatment of infections in the human respiratory tract, urinary tract, kidney, and gastrointestinal system, and for other bacterial infections.^76,225^ Retransformation of *N*-AcSMX and SMX-*N*-glucuronide back to SMX was reported in some studies, and thus these TPs could be extra sources of active sulfonamides within the environment after hydrolysis of the glucuronide.^74,193,223,226^ For SMX-*N*-glucuronide, reformation was suggested to occur in the recipient water body, due to the weak glucuronide bond. For *N*-AcSMX, reformation was suggested to occur either in the wastewater treatment facility or due to sediment–water interactions in the environment.^223^ The concentrations of *N*-AcSMX in surface waters were found to range up to 270 ng L^–1^ in several countries.^221^ Brenner et al.^76^ and Kokoszka et al.^206^ applied suspect screening to detect *N*-AcSMX. Kokoszka et al.^206^ further identified various sulfonamide TPs, including five (a)biotic environmental and human TPs of SMX and four TPs of sulfadiazine, in recipient water bodies. Their findings suggest that the fate of the parent compound sulfapyridine might be of interest for selective pressure assessment because it was indicated to be environmentally stable due to the lack of human and environmental TPs detected.

Similarly to SMX, *N*-acetylated TPs have also been detected for sulfadiazine (in concentrations up to 92 ng L^–1^),^78,198−202^ sulfamerazine (420 ng L^–1^),^79,208,209^ sulfamethazine (695 ng L^–1^),^78,131,199,200,203−206^ and sulfapyridine (133 ng L^–1^).^79^ Cui et al.^79^ quantified various *N*-acetylated TPs as the predominant species in surface waters and also detected three minor TPs corresponding to SMX and sulfapyridine (4-nitrososulfamethoxazole, 5-hydroxysulfapyridine, and 5-[4-(acetylamino)benzenesulfonyloxy]sulfapyridine), in concentration ranges of 0.1–7.1, 0.3–9.2, and 0.2–3.3 ng L^–1^, respectively.

#### 5.2.2 Macrolides

Of 75 articles reviewed, 19^106,187−204^ detected 8 different macrolide (bio)TPs in surface waters (Table S10). TPs from the parent compound erythromycin (ERY)^104,179,179,181−196^ were the most frequently reported (95%, 18 of 19 articles). A total of eight TPs representing three classes of macrolides were reported (Table S10). Anhydro-ERY was the most commonly studied.

Within the macrolide family, the papers reviewed most often studied TPs of ERY in surface waters, especially its human bioTP, anhydro-ERY, which is formed via dehydration under the acidic conditions in the stomach.^219−221^ ERY is a narrow-spectrum antibiotic that is effective against specific families of bacteria, whereas other macrolides, e.g., azithromycin, are broad-spectrum antibiotics. Erythromycin is used to treat, e.g., respiratory tract infections, skin infections, chlamydia infections, and syphilis.^230^ Anhydro-ERY was detected in concentrations within the range 0.13–10000 ng L^–1^ in studies on different surface waters (Table S10). Senta et al.^187^ demonstrated the significant contribution of human metabolites to the overall mass balance of ERY and other macrolides in aquatic environments, due to poor removal efficiencies for both parent and human TPs.^231^ Anhydro-ERY was suggested by one study to no longer exhibit antibiotic properties.^232−235^ ERY A enol ether, another human bioTP of ERY, was detected by Mokh et al.^190^ in surface water, at concentrations of 20–780 ng L^–1^. ERY A enol ether was found to be in equilibrium with ERY, while ERY is directly converted into anhydro-ERY.^225−229^ Steinmetz et al.^236^ investigated ERY A enol ether mimicking properties of the intestinal peptide hormone motilin due to structural similarities, which could lead to gastrointestinal complaints.

Only two studies^187,188^ determined TPs of other macrolides, azithromycin and clarithromycin, in surface waters (Table S10). Senta et al.^187^ detected descladinosyl azithromycin, *N*′-desmethyl azithromycin, and phosphorylated azithromycin at concentrations of up to 5300, 8600, and 860 ng L^–1^, respectively. For clarithromycin TPs, Senta et al.^187^ detected *N*′-desmethyl clarithromycin at 2000 ng L^–1^, and Baumann et al.^188^ detected 14-hydroxyclarithromycin at 80 ng L^–1^. Although these concentrations are below the MICs reported by Martin et al.,^237^ potential synergistic effects of parent and metabolite could pose a risk of emergence and proliferation of resistance genes.

Macrolide TPs can also be created during the manufacture of macrolide antibiotics. To date, two synthesis TPs (byproducts) have been reported in surface waters (Table S10).^187^ One is *N*-desmethyl azithromycin, which is a synthesis intermediate of azithromycin, while the other is ERY oxime. In a Croatian surface water environment downstream of an industrial discharge point, Senta et al.^187^ measured *N*-desmethyl azithromycin in concentrations of 5500–8600 ng L^–1^ and ERY oxime in concentrations of 1300–19 000 ng L^–1^. Several ERY-oxime analogues have been shown to exhibit similar antibacterial activity to ERY.^238^

#### 5.2.3 β-Lactams

Five of the studies reviewed detected nine different TPs corresponding to two β-lactam classes, amoxicillin and benzylpenicillin (penicillin G). Amoxicillin is one of the most widely used penicillin antibiotics, to treat, e.g., pneumonia, pharyngitis, and urinary tract infections.^239^ Benzylpenicillin is used to treat, e.g., pneumonia, syphilis, diphtheria, cellulitis, and tetanus.^240^ Li et al.^186^ investigated the fate of benzylpenicillin and five TPs in river water receiving effluent discharged from a production facility of the North China Pharmaceutical Group Corporation and recorded elevated concentrations of five TPs (isopenillic acid, benzylpenilloic acid, benzylpenicilloic acid, benzylpenillic acid, and benzylpenicilloaldehyde) at concentrations up to 0.94, 11, 1.8, 1.2, and 1.3 mg L^–1^, respectively. The five TPs tended to be persistent in the water body, as the decline in their concentrations was comparatively small over 30 km, and benzylpenilloic acid was the dominant TP, accounting for over 60% of the TP contamination profile in the river water.^186^

Four studies^101−104^ reported four AMX TPs (Table S10). Angeles et al.^106^ detected AMX penicilloic acid and penilloic acid in concentrations of 7.4 and 246 ng L^–1^, respectively. These two TPs, together with 3-(4-hydroxyphenyl)pyrazinol and AMX 2′,5′-diketopiperazine, were also detected (without quantification) by Goessens et al.,^104^ Pérez-Parada et al.,^105^ and Hirte et al.^103^ Microbial activity of the AMX TPs is likely reduced due to opening up of the β-lactam ring.^241^

#### 5.2.4 Lincosamide

Only one study detected a lincosamide TP within the clindamycin class (Table S10). Clindamycin is mainly used to treat anaerobic infections, including dental and respiratory tract infections.^242^ Boulard et al.^162^ recorded 120 ng L^–1^ clindamycin sulfoxide in river water from Germany. This clindamycin TP was suggested to be persistent in the aquatic environment.^243^

#### 5.2.5 Tetracyclines

Five studies^104,197,207,219,244^ detected seven different TPs of three tetracycline classes, chlortetracycline, oxytetracycline, and tetracycline itself (Table S10). Tetracyclines are broad-spectrum antibiotics that exhibit activity against a wide range of microorganisms, including Gram-positive and Gram-negative bacteria, and protozoan parasites.^245^ Tetracycline TPs were more commonly studied than TPs of chlortetracycline and oxytetracycline in the papers reviewed here. Among tetracycline TPs, 4-epitetracycline was the most reported and quantified in a range of 11.5–9210 ng L^–1^ in Belgium by Goessens et al.,^104^ in China by Jiang et al.,^207^ and in Turkey by Topal and Arslan Topal.^219,220^ Topal and Arslan Topal^219,220^ also reported high concentrations of two other tetracycline TPs, the anhydro-derivates 4-epianhydrotetracycline (at 6.8–37.2 μg L^–1^) and anhydrotetracycline (at 4.4–6.4 μg L^–1^). Anhydro-derivate TPs of tetracyclines were found to have strong embryotoxic and teratogenic properties,^246^ posing a potential risk to nontarget organisms in aquatic environments. While TPs are generally believed to be less microbiologically active, anhydrotetracycline had an EC_50_ value for selected bacteria that was approximately three times lower than that of the parent tetracycline.^247,248^ For chlortetracycline TPs, Goessens et al.^104^ and Chang et al.^197^ recorded 4-epichlortetracycline and isochlortetracycline in concentrations of up to 84.4 and 15 ng L^–1^, respectively. The oxytetracycline TP 4-epioxytetracycline was quantified by Goessens et al.^104^ and Jiang et al.^207^ in concentrations of 3.5–84.9 ng L^–1^.

#### 5.2.6 Nitroimidazoles

The only reported TP of nitroimidazoles in surface waters was hydroxymetronidazole (Table S10), which is the active metabolite of metronidazole (MTZ^249,250^). Metronidazole is used to treat, e.g., pelvic inflammatory disease, endocarditis, and bacterial vaginosis.^251,252^ It was found in concentrations from 65 to 11 300 ng L^–1^ in studies in Spain. Furthermore, the active metabolite was detected in higher concentrations than the parent MTZ. Studies have shown that hydroxymetronidazole is 10 times more potent than MTZ, based on the Ames test for mutagenicity with *Salmonella typhimurium* TA1535.^253,254^ Human urinary isolates of MTZ and its metabolite have been found to increase gene mutations in bacteria.^255^

### 5.3 Other Antibacterials

Nine studies^130,133−138,250,251^ investigated five TPs corresponding to antibacterial agents triclosan and triclocarban in surface waters (Table S11). Triclosan and triclocarban are used as antimicrobial agents in various nursing products and as disinfectants in personal care products. Methyl triclosan was the most studied TP (8 of the 9 studies) and was quantified at 0.006–191 ng L^–1^. Coogan et al.^257^ reported a potential for bioaccumulation of methyl triclosan in biota in water streams receiving effluent wastewater, due to its higher stability and lipophilicity compared with triclosan. For triclocarban, three TPs were determined: carbanilide,^142,256^ dichlorocarbanilide,^142,256^ and 1,3-bis(3,4-dichlorophenyl)urea,^256^ in concentrations up to 67–188, 2–615, and 615 ng L^–1^, respectively. These three TPs, formed via reductive dechlorination, were linked to endocrine disruption.^258,259^ In addition, triclosan can be converted into 2,8-dichlorodibenzo-*p*-dioxin in environmental waters via photolysis.^260^ Although induced stress from 2,8-dichlorodibenzo-*p*-dioxin was not found in several bacterial strains tested,^261^ it was suggested to have endocrine-disrupting effects on mammals and aquatic organisms.^262,263^

## 6 Risk and Hazard Evaluations of TPs

### 6.1 Antibiotic Resistance Risk

While PNEC_AMR_ values relating to induced selection pressure on bacteria are available for several parent antibiotics,^52,264^ there is little to no knowledge on PNEC_AMR_ for their TPs. Considering the role of chemical structure in promoting the growth of resistant bacteria, we evaluated the structural similarity between TPs and their respective parent compounds using 3D and 2D measures, as prior knowledge to performing risk evaluation by RQ_AMR_ (eqs 1 and 2). Of the 56 TPs compiled in this review, 14 showed high similarity to the respective parent compound (Table S2). Five of these, 4-epitetracycline, *N*-desmethyl azithromycin, anhydro-ERY, anhydrotetracycline, and ERY oxime, displayed a risk of inducing resistance development in the environment, with RQ_AMR,similar_ values of up to 34. Some TPs with low similarity to parent compounds still had high RQ_AMR,dissimilar_, including 4-epianhydrotetracycline (RQ_AMR,dissimilar_ = 3.7), descladinosyl azithromycin (2.1), hydroxymetronidazole (9.1), and five benzylpenicillin TPs (benzylpenicilloaldehyde (520), benzylpenicilloic acid (720), benzylpenillic acid (480), benzylpenilloic acid (4200), and isopenillic acid (370)). The high RQ_AMR,dissimilar_ for all benzylpenicillin TPs was influenced by the fact that their measured concentrations were several orders of magnitude higher than those of all other TPs detected, even though their active moiety is generally considered to lose its pharmacological activity after hydrolysis (ring opening). Four TPs of high similarity (14-hydroxyclarithromycin, 4-epioxytetracycline, clindamycin sulfoxide, and ERY A enol ether) were close to triggering an AMR risk with 0.1 < RQ_AMR,similar_ < 1. Two dissimilar TPs, *N*′-desmethyl clarithromycin and phosphorylated azithromycin, also showed 0.1 < RQ_AMR,similar_ < 1. Overall, 13 TPs had RQ_AMR_ > 1, six had 0.1 < RQ_AMR_ < 1, and 13 had RQ_AMR_ < 0.1 (Tables S2 and S13). We estimated RQ_AMR_ of the respective parent compounds based on their MEC within our literature review. Most TPs showed similar RQ_AMR_ as the respective parent (Table S13), with the exceptions of tetracycline and metronidazole TPs with higher AMR risk than the parent compounds and amoxicillin TPs with lower AMR risk than the parent compound. Of the 56 detected TPs, 24 could not be assessed because PNEC_AMR_ values for the parent compounds were not available (Table S2 and S13). Our approach for obtaining RQ_AMR_ of TPs based on PNEC_AMR_ of the respective parent compounds after structural similarity evaluations remains conservative but helps fill current knowledge gaps on understanding AMR risks attributable to antimicrobial TPs.

### 6.2 Ecological Risk

Experimental EC_50_ values for the parent compounds were collected from the literature (Table S3). We used QSAR-based EC_50,baseline_ predictions (eqs 3–5) to calculate toxic ratios of the parent TR(P) (eq 8, illustrated in Figure S3a). The challenge is that antibiotics are often ionizable and rather hydrophilic, so the conventional *K*_ow_-based QSARs are not valid. However, there is an empirical log *D*_lipw_-based QSAR for *A. fischeri* (eq 3),^59^ while the QSARs for other species (eqs 4 and 5) were adapted to ionizable chemicals by rescaling from *K*_ow_ to *D*_lipw_.^53,54^

Although the 30 min bioluminescence inhibition test with *Aliivibrio fischeri* is only a poor descriptor of bacterial toxicity and much less sensitive that bacterial growth inhibition assays over 24 h,^265^ it remains the most data-rich screening assay with bacteria. Most antimicrobials showed excess toxicity, with TR > 10, and clindamycin had the highest TR (14000) (Figures S3b and S4a and Table S3). The nontarget species *Pseudokirchneriella subcapitata* and *Daphnia magna* were also substantially affected, with TRs ranging up to 65000 for *P. subcapitata* and 63 for *D. magna* (Figures S3c–d and S4a and Table S3). Green algae had similar TR ranges as *A. fischeri*, while antimicrobials acted less specifically on *D. magna* (Figure S4a and Table S3).

As no toxicity data were available for the TPs, we estimated EC_50,baseline_ of the TPs and used the TR value of the parent compound for TPs with similar structure (eq 9) and a TR value of TR(P)/10 for TPs with dissimilar structure (eq 10) to estimate EC_50_ of the TPs. This is illustrated exemplarily for clarithromycin in Figure S5. The distribution of TR(TP) was skewed toward lower TR values, but the TPs still covered a wide range of specificity due to similarity to the parent compound (Figure S4b). Similarly to the parent antimicrobials, a lower specific toxicity toward *D. magna* was observed. Methyl triclosan displayed the highest toxicity, that is, the lowest EC_50_ toward *A. fischeri* (1.4 × 10^–9^ mol L^–1^) and *D. magna* (3.0 × 10^–7^ mol L^–1^), and 14-hydroxyclarithromycin displayed the lowest EC_50_ toward *P. subcapitata* (6.5 × 10^–9^ mol L^–1^, Figure S5a–c and Table S3).

**Figure 3:**
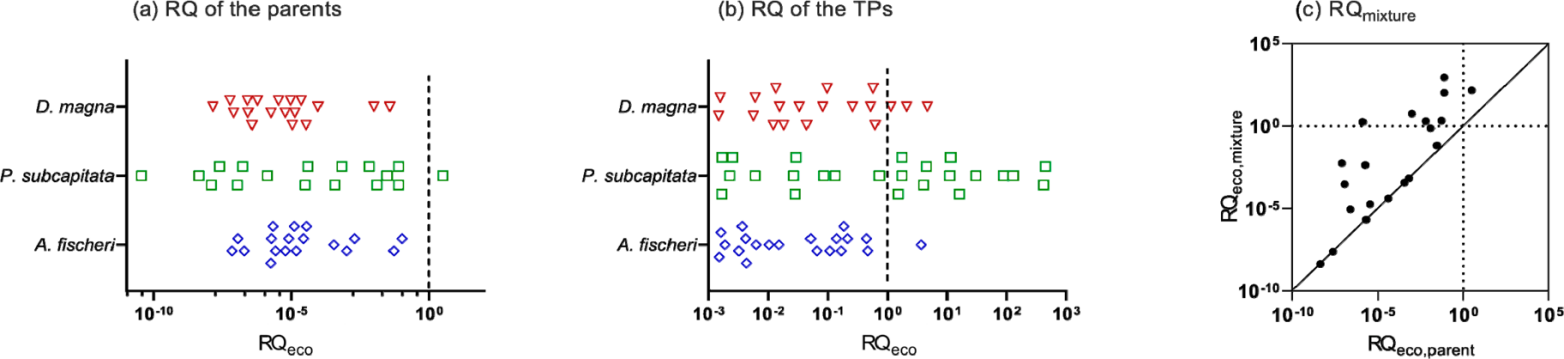
Range of risk quotients (RQ_species_) of the parent compound (a) and TPs (b) covered by the literature included in this review; (c) comparison of RQ_eco,parent_ with RQ_eco,mixture_ (eq 14).

The RQs were calculated using the MEC values (Table S3), and most parents displayed a RQ_species_(P) < 1. Only benzylpenicillin had a RQ_*P.subcapitata*_(P) > 1 (Figure 3a, Tables S3 and S13). For the TPs, only benzylpenicilloaldehyde had a RQ_species_(TP) > 1 for all three investigated species (Figure 3b). With few exceptions (hydroxymetronidazole, oseltamivir, sulfapyridine and its TPs), the RQ_*A.fischeri*_ was lower than RQ_*P.subcapitata*_. Seven TPs (clindamycin sulfoxide, anhydro-ERY, ERY oxime, benzylpenillic acid, isopenillic acid, 1,3-bis(3,4-dichlorophenyl)urea, and methyl triclosan) were close to triggering a risk with 0.1 < RQ_*A.fischeri*_ < 1. Thirteen TPs showed a risk to at least one of the nontarget species (*P. subcapitata* and *D. magna*). Six of these TPs belonged to the parent class of the macrolides, three to β-lactams, three to phenoxyphenols and one to antiviral.

The combined risk of parent and TP, RQ_eco,mixture_, was generally higher than the corresponding RQ_eco,parent_ (Figure 3c, Table S3). Only one antibiotic (penicillin G) had a RQ_eco,parent_ > 1, but RQ_eco,mixture_ exceeded 1 for additional 6 antimicrobials. This emphasizes the importance of TPs for the ecological risk assessment. Since most estimated effect concentrations (EC_50_) of the TPs were either in the same range or even higher than the respective parent compound (lower toxicity), the higher RQ_eco,mixture_ can be explained by the higher environmental concentrations of TPs as compared to the parent. For example, the RQ_eco,mixture_ of efavirenz was predicted to be >1 due to the high MECs of 8,14-dihydroxyefavirenz, despite its dissimilarity to its parent efavirenz, and the PNEC_*P.subcapitata*_ was 660 times higher for the TP than for the parent. This observation is supported by the increased persistence estimate of several TPs compared to their parent compounds (see section 6.4.1). Almost all TPs showing a risk for resistance development also posed a risk to at least one of the three species.

### 6.3 Human Health Hazard Evaluation

#### 6.3.1 Mutagenicity

Mutagenicity refers to an increase in the mutation rate via different pathways, including nucleotide-pool unbalancing and general stress responses such as production of reactive oxygen species that cannot be removed by repair mechanisms. Mutations are a major mechanism for the development of antibiotic resistance genes in bacteria.^266^ Therefore, organisms exposed to low concentrations of mutagenic antimicrobial TPs may be subject to antimicrobial-induced mutation and recombination hotspots, which are responsible for phenotypic variation and specifically for the proliferation and dissemination of resistance genes.^267^

The model performance parameter showed a good correlation (MCC = 0.5–0.6) for both models used. An increase in certainty of predicted mutagenic activity (consensus score) compared to the respective parent was observed for six TPs (Tables S7 and S13), namely, anhydrotetracycline, 4-epianhydrotetracycline, 4-epitetracycline, hydroxymetronidazole, sulfamethoxazole beta-d-glucuronide, and 3-(4-hydroxyphenyl)pyrazinol. Thus, tetracycline TPs were more often mutagenic. Six TPs (carboxy-abacavir, carboxy-acyclovir, 4-epichlortetracycline, clindamycin sulfoxide, 4-epioxytetracycline, and apo-oxytetracycline) showed similar predicted mutagenic activity as the respective parent compound. For isochlortetracycline, 14-hydroxyclarithromycin, *N*′-desmethyl clarithromycin, and *N*-acetylsulfamethazine, the predicted mutagenic activity was lower than that of the respective parent. Lv et al.^268^ found a correlation between mutagenicity and resistance development for halogenated nitrogenous disinfection byproducts. Further investigation is needed, as resistance-inducing mechanisms may not only be attributable to antimicrobial substances but also to the environmental consequences of compound mutagenesis.^269^

#### 6.3.2 Carcinogenicity

Among the 56 TPs listed, 11 showed increased, 10 similar, and 11 decreased predicted carcinogenic potentials compared with the respective parent compound (Tables S7 and S13), while the remaining 24 showed no predicted carcinogenicity. Of the 11 TPs with increased carcinogenicity, almost half (*n* = 7) belong to the sulfonamide class. Three different antiviral TPs (carboxy-abacavir, 8,14-dihydroxyefavirenz, and emtricitabine S-oxide) and one macrolide TP (ERY A enol ether) also displayed an increase in carcinogenicity. Kilkkinen et al.^270^ found an association between antibiotic use and increased risk of cancer in a Finnish cohort study. Some antibiotics have been found to promote tumor development.^271,272^

### 6.4 Environmental Hazard

#### 6.4.1 Persistence

The persistence of the TPs was evaluated using the VEGA model (Table S8). Of the 56 TPs, 14 were considered persistent (degradation half-life >40 days) according to the REACH guideline. Eight of these belonged to the macrolide family (14-hydroxyclarithromycin, *N*-desmethyl azithromycin, *N*′-desmethyl clarithromycin, anhydro-ERY, descladinosyl azithromycin, ERY A enol ether, ERY oxime, and phosphorylated azithromycin), one was a lincosamide TP (clindamycin sulfoxide), two were tetracycline TPs (4-epichlortetracycline and apo-oxytetracycline), one was a sulfonamide TP (SMX beta-d-glucuronide), and two were antiviral TPs (8,14-dihydroxyefavirenz and oseltamivir carboxylate). Most TPs were in the same persistent range as the respective parent compound (Table S13). Oxytetracycline persistence was predicted to be one-third that of the TP (apo-oxytetracycline). This is in line with previous findings on tetracycline dissipation in semifield microcosm conditions.^94^ Similarly, 8,14-dihydroxyefavirenz and SMX-beta-d-glucuronide showed high persistence, which was not given for the respective parents, efavirenz and SMX.

#### 6.4.2 Mobility

In the proposed revision of the Classification, Labeling and Packing (CLP) Regulation, new criteria for assessing chemical mobility are envisaged to be included.^273^ The binding constant to organic carbon is the measure to quantify mobility in water with a proposed threshold of log *K*_OC_ < 3. However, recent developments suggest that the *K*_OC_ threshold should be increased to log *K*_OC_ ≤ 4.5 to account for differences in the mobility of ionizable chemicals at different pH values, although this has not yet been implemented in legislation.^274^ For the purpose of this review, we used solubility >150 μg L^–1^ and log *K*_OC_ ≤ 4.5 as mobility criterion. Almost all TPs (50 of 56) were classified as mobile (Table S8). *N*-Desmethyl azithromycin, *N*′-desmethyl clarithromycin, ERY A enol ether, ERY oxime, methyl triclosan, and phosphorylated azithromycin were not sufficiently water-soluble or had too high of a *K*_OC_ value to be mobile. All respective parent antimicrobials to the immobile TPs, except methyl triclosan, belong to the class of macrolides and are of higher molecular weight than other antibiotic classes.

#### 6.4.3 Bioconcentration Factor

Only two of the 56 TPs, namely, the neutral TPs, 1,3-bis(3,4-dichlorophenyl)urea and methyl triclosan, exceeded the REACH threshold^275^ of log BCF ≤ 3.3, with values of 3.3 and 3.7, respectively (Table S8). This result appears reasonable, as most antimicrobials are polar organic chemicals and transformation processes mostly lead to even more polar TPs. For example, the BCF of clindamycin (1.09) is about 1 order of magnitude higher than that of its TP clindamycin sulfoxide (0.5) (Table S13). Overall, BCF appears to play a minor role in the environmental hazard of antimicrobial TPs (Table S8).

## 7 Prioritization of TPs of Concern

**Figure 4:**
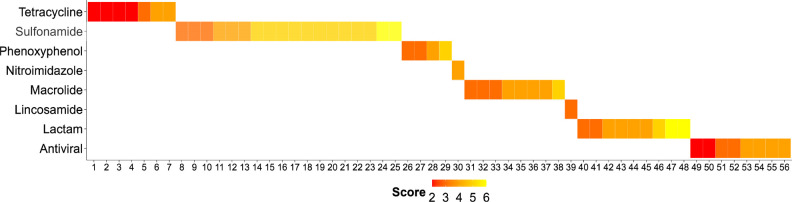
Scored antimicrobials (TPs, *n* = 56) grouped according to the chemical class of the parent compound. The lower the score, the higher the degree of concern. Tetracycline TPs: (1) 4-epianhydrotetracycline, (2) 4-epichlortetracycline, (3) 4-epitetracycline, (4) anhydrotetracycline, (5) apo-oxytetracycline, (6) 4-epioxytetracycline, (7) isochlortetracycline; sulfonamide TPs: (8) 5-hydroxysulfadiazine, (9) *N*-acetylsulfamethazine, (10) 5-hydroxysulfapyridine, (11) SMX beta-d-glucuronide, (12) *N*-acetylsulfadiazine, (13) *N*-acetyl-SMX, (14) 4-formamido-*N*-(2-pyrimidinyl)benzenesulfonamide, (15) 4-*N*-methyl-SMX, (16) 4-amino-*N*-[(1*E*)-1-amino-3-oxobut-1-en-1-yl]-2-hydroxybenzene-1-sulfonamide, (17) 4-amino-*N*-[(1*E*)-1-amino-3-oxobut-1-en-1-yl]benzene-1-sulfonamide, (18) 4-amino-*N*-methylbenzenesulfonamide, (19) 4-nitroso-SMX, (20) 5-[4-(acetylamino)benzenesulfonyloxy]sulfapyridine acetate, (21) *N*-acetylsulfamerazine, (22) *N*-acetylsulfapyridine, (23) benzenesulfonic acid, (24) *N*-dimethyl-SMX, (25) carboxy-SMX; phenoxyphenol TPs: (26) methyl triclosan, (27) 1,3-bis(3,4-dichlorophenyl)urea, (28) dichlorocarbanilide, (29) carbanilide; nitroimidazole TP: (30) hydroxymetronidazole; macrolide TPs: (31) *N*-desmethyl azithromycin, (32) anhydro-ERY, (33) descladinosyl azithromycin, (34) 14-hydroxyclarithromycin, (35) *N*′-desmethyl clarithromycin, (36) ERY A enol ether, (37) ERY oxime, (38) phosphorylated azithromycin; lincosamide TP: (39) clindamycin sulfoxide; β-lactam TPs: (40) benzylpenillic acid, (41) isopenillic acid, (42) 3-(4-hydroxyphenyl)pyrazinol, (43) benzylpenicilloaldehyde, (44) benzylpenicilloic acid, (45) benzylpenilloic acid, (46) AMX penilloic acid, (47) AMX penicilloic acid, (48) AMX-diketopiperazine-2′5′; antiviral TPs: (49) 8,14-dihydroxyefavirenz, (50) carboxy-abacavir, (51) carboxy-acyclovir, (52) emtricitabine *S*-oxide, (53) 12-hydroxynevirapine, (54) carboxy-emtricitabine, (55) carboxy-lamivudine, (56) oseltamivir carboxylate.

To prioritize all 56 TPs of this review in terms of degree of concern, the risk and hazard parameters were scored according to all criteria (Table 1), followed by ranking based on these scores (Table S8 and Table 2). There were six TPs (Table 2) with the lowest score (2), of which four were TPs of tetracyclines (4-epianhydrotetracycline, 4-epichlortetracycline, 4-epitetracycline, and anhydrotetracycline) and two were TPs of antivirals (8,14-dihydroxyefavirenz, carboxy-abacavir). The 14 TPs with the second lowest score (3) belonged to macrolides, sulfonamides, antivirals, β-lactams, phenoxyphenols, lincosamides, and tetracycline (Figure 4 and Table S8). Most of the β-lactam TPs had a final score of 4–6, meaning less concern, which is consistent with the fact that their pharmacological activity is known to be reduced by opening up of the β-lactam ring moiety.^241^ Exceptions were the β-lactam TPs, benzylpenillic acid, and isopenillic acid, with a final score of 3, which was attributable to their RQ_AMR,dissimilar_, RQ_species_, carcinogenicity, and mobility values (Table S8). The final score (2) of the top six TPs is lower than that of their respective parent compounds (final scores of 3–5) (Table S13), meaning that these TPs are not only the most concerning among the compiled TPs but also of higher concern than their respective parent compounds.

**Table 2:** Top Six Antimicrobial TPs of Concern (See Table S8 for the Full Ranking List and Table S13 for Comparison between Some TPs and Parent Compounds)^a^

		**Scoring**							
**Antimicrobial TP**	**Respective parent family**	**AMR risk**	**Eco risk**	**M**	**C**	**P**	**BCF**	**M′**	**Final**
4-Epianhydrotetracycline	Tetracycline	0	0	0	0	1	1	0	2
4-Epichlortetracycline	Tetracycline	0	0	1	1	0	1	0	2
4-Epitetracycline	Tetracycline	0	0	0	0	1	1	0	2
8,14-Dihydroxyefavirenz	Antiviral	1	0	1	0	0	1	0	2
Anhydrotetracycline	Tetracycline	0	0	0	0	1	1	0	2
Carboxy-abacavir	Antiviral	1	0	0	0	1	1	0	2

a AMR = antimicrobial resistance; Eco = ecological; M = mutagenicity; C = carcinogenicity; P = persistence; BCF = bioconcentration factor; M′ = mobility.

As mentioned in section 6.1, it was not possible to obtain RQ_AMR_ for 24 TPs, due to a lack of available data on PNEC_AMR_ of the parent compounds, and these were allocated a score of 0. At the minimum, the final score for these TPs thus presented a concern and could be updated in future if PNEC_AMR_ data for the parent compounds become available.

The TPs of most concern came from different antimicrobial families (Figure 4). In general, tetracycline (scores of 2–4) and antiviral TPs (scores of 2–4) found in surface waters were of higher concern than most sulfonamide TPs (scores of 3–6) (Figure 4). It is important to note that although many studies excluded the direct toxic effects of specific compounds (e.g., TPs) on selected indicator species at environmentally relevant concentrations, the mixture toxicity and influence on the food web of micro- and macrosystems should be considered.

## 8 Remarks for the Future

**Figure 5:**
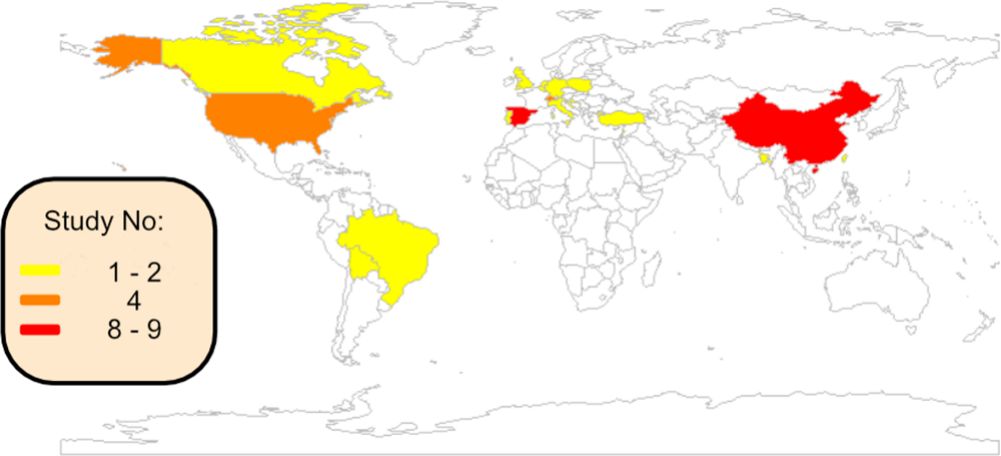
Number of studies per country detecting antibiotic TPs in surface waters. See Figure S2A for antiviral TPs and Figure S2C for TPs of other antibacterials.

Antimicrobial TPs are an overlooked chemical class compared to TPs of other chemicals.^44^ The earliest study reporting antimicrobial TPs^136^ was published about two decades ago, which time-wise aligned with the development and usage of high-resolution mass spectrometry, which is necessary for identification.^276,277^ We observed a clear geographic difference in the available data for antibiotic, antiviral, and other antibacterial TPs (Figures S2A–C). For antibiotic TPs (Figures 5 and S2B), data gaps exist for Africa, Oceania, most of South America and Asia. There were even fewer data on TPs of antivirals and other antibacterials (Figures S2A and C). Reported detections were mainly from Europe and sporadically from Asia (only Japan) and Africa (South Africa). The lack of standardization in monitoring antimicrobial chemicals and AMR has been recently pointed out elsewhere.^278^

The analysis of existing literature and the simple screening approach to include the risk of TPs that was provided here may serve for predictions of antimicrobials’ risk. As our review only included the studies with detectable TPs in surface waters, the possibility remains that TPs may be present at concentrations below the limit of detection in these and other countries. For example, in 2015 Algeria had a similar rate of antibiotic use (14000 defined daily doses per 1000 inhabitants)^279^ as well-studied countries such as Spain or Turkey, but no TP studies have been performed on antibiotic TPs in Algeria, whereas TPs of several antibiotic classes have been reported in Spain and Turkey. A recent study investigating a wide range of pharmaceutical parent compounds found even more environmental pollution in low- and middle-income countries than in better-studied high-income countries.^280^ Given that extensive analysis of TPs in surface water is not always feasible and could be cost-prohibitive for many low- and middle-income countries, we recommend considering consumption data on antimicrobial chemicals to preliminarily estimate the occurrence and risk of the TPs.

The TPs covered by this review corresponded to parent antimicrobial compounds, half of which are listed as essential and last-resort medicines by the WHO.^281^ While the relationship between the parent antimicrobial compounds and AMR is well documented, the impacts of their TPs on AMR development (through alternative or enhanced selective pressure on resistant bacteria) and on environmental health are not well understood. Our ranked list of 56 TPs indicates that many TPs are of global concern in surface water environments and especially the top six TPs that exceed the hazard or risk thresholds for 5 of 7 assessed categories (Table 2). Future action on these TPs is warranted, such as regulation of their discharge and that of the corresponding parent compounds to the environment, reducing usage of the parent compounds, and improving removal efficiency through advanced wastewater treatment techniques. Including TPs in risk assessments of the parent compounds would increase the safety of new antimicrobial chemicals developed and marketed in the future.

Another future research requirement is to determine MICs, and therefore PNEC_AMR_, for antimicrobial TPs. These are so far unavailable for most TPs, preventing more realistic risk assessment of RQ_AMR_ values. Our workflow partly helped to overcome this limitation by providing new insights into RQ_AMR_ values of TPs based on their structural similarity to parent compounds and the use of parent PNEC_AMR_ values.

Furthermore, the bioavailability of antibiotics and TPs is often overlooked and requires further investigation. The term itself is defined differently in different research fields and for different target organisms.^282^ In this review article, we assumed that the bioavailability of an antibiotic or TP is the fraction that causes selection pressure on the target bacteria, although that leaves unanswered the question of which species are the target bacteria. Bioavailability is naturally affected by the biological, chemical, and physical conditions of the living environment of the bacteria. Approaches for measuring the bioavailability of various compounds, including antibiotics, have been developed by using chemical methods connected with different extraction methods mimicking the biology, e.g., by Jimenez et al.^283^ Another approach for assessment of bioavailability is the use of genetically engineered bacteria (bioreporters),^284^ but no substantial breakthrough has been made in this area. Measuring the actual selection pressure, i.e., the effect of a compound on bacterial growth, is perhaps still the best method and there are different options available, such as using a single bacterial species or a microbial community.^285^ It is not unreasonable to claim that understanding bioavailability will be a major focus of research in coming years.

We calculated RQ_AMR_ and RQ_species_ for all TPs individually. Several of the TPs covered by this review were found to pose an ecological risk RQ_eco_ to the surface waters. The reality is that TPs coexist with their parents and that they are likely to act together in mixtures, especially those with similar structures. The RQs of parent and TP can be simply summed up (eq 13). Although the mixture risk quotient of parent and TP was generally not much higher than the RQ of the TP, this analysis is only preliminary because it is based exclusively on predicted effect data. More data on global environmental occurrence and experimental toxicity data of TPs would facilitate the mixture toxicity assessment and management of antimicrobials to ultimately achieve sustainable surface water environments.

## Supporting Information Available

Systematic literature search flow diagram, number of studies that detected or quantified TPs in surface waters, theoretical difference between predicted baseline toxicity and specific toxicity endpoint, range of toxic ratios, and estimated specific toxicity of clarithromycin and its TPs (PDF)
Summary of selected literature; similarity evaluation and RQ_AMR_ calculations; EC_50_, PNEC, and RQ estimations of the compiled TPs; database information on mutagenicity and carcinogenicity; mutagenicity and carcinogenicity literature of parent compounds; parameters assessing model performance of mutagenicity and carcinogenicity predictions; mutagenicity and carcinogenicity estimations of compiled TPs and respective parent compounds; TPs with characterization parameters and scores for prioritization; antiviral TPs detected and quantified in surface waters; detected antibiotic TPs together with their estimated form as well as concentration; other antibacterial TPs detected in surface waters; information about associated antiviral parent compounds; and comparison of RQ_AMR_, RQ_species_, mutagenicity, carcinogenicity, persistence, mobility (solubility and log *K*_OC_), and log BCF between TPs and parent compounds (XLSX)
