# Supplementary material for: Antimicrobial Transformation Products in the Aquatic Environment: Global Occurrence, Ecotoxicological Risks, and Potential of Antibiotic Resistance
Source: Environ Sci Technol. 2023 Jun 19;57(26):9474–94. doi: 10.1021/acs.est.2c09854 (PMC10324322; doi:10.1021/acs.est.2c09854)
Supplement: Supplementary file 1 — es2c09854_si_001.pdf [file es2c09854_si_001.pdf]

Supporting Information

Antimicrobial Transformation Products in the Aquatic Environment: Global Occurrence, Ecotoxicological Risks and Potential of Antibiotic Resistance

Paul Löffler[1,*] Beate I. Escher[2,3], Christine Baduel[4], Marko P. Virta[5,6], Foon Yin Lai[1,*]

[1] Department of Aquatic Sciences and Assessment, Swedish University of Agricultural Sciences (SLU), Uppsala, SE-75007, Sweden

[2] Department of Cell Toxicology, Helmholtz Centre for Environmental Research, UZ, 04318 Leipzig, Germany

[3] Eberhard Karls University Tübingen, Environmental Toxicology, Department of Geosciences, 72076 Tübingen, Germany

[4] Université Grenoble Alpes, IRD, CNRS, Grenoble INP, IGE, 38 050 Grenoble, France

[5] Department of Microbiology, Faculty of Agriculture and Forestry, University of Helsinki, 00014 Helsinki, Finland

[6] Multidisciplinary Center of Excellence in Antimicrobial Resistance Research, Finland

[*]Corresponding authors [emails]

Paul Löffler [paul.loffler@slu.se]

Foon Yin Lai [foonyin.lai@slu.se]

Table of Contents

Figure S1: Systematic literature search flow diagram with inclusion and exclusion criteria.

Figure S2: Number of studies that detected or quantified TPs in surface waters for antivirals (A), antibiotics (B), and other antibacterials (C).

Figure S3: (a) Theoretical difference between predicted baseline toxicity and specific toxicity endpoint; parent antimicrobial toxicity towards (b) *A. fischeri*, (c) *P. subcapitata*, and (d) *D. magna* including baseline toxicity prediction (line) using Equations 3 to 5.

Figure S4: Range of toxic ratios TR of (a) the parent antibiotics and (b) the transformation products TP.

Figure S5: Estimated specific toxicity of clarithromycin and its TPs (a,b,c) towards *A. fischeri* (a,d), *P. subcapitata* (b,e), *D. magna* (c,f).

Table S1: Summary of selected literature, identified transformation products (TPs) in surface waters, respective compound class, family and parent compound of the TPs, TP concentration range, instrumentationand country of the studied surface water (excel sheet).

Table S2: Similarity evaluation and RQ_{AMR} calculations (excel sheet).

Table S3: EC_{50}, PNEC and RQ estimations of the compiled TPs (excel sheet).

Table S4: Database information on mutagenicity and carcinogenicity to validate predictions (excel sheet).

Table S5: Mutagenicity and carcinogenicity literature of parent compounds to validate the prediction (excel sheet).

Table S6: Parameters assessing model performance of mutagenicity and carcinogenicity predictions (excel sheet).

Table S7: Mutagenicity and carcinogenicity estimations of compiled TPs and respective parent compounds (excel sheet).

Table S8: TPs with characterization parameters and scores (light grey filled) for prioritization (excel sheet).

Table S9: Antiviral TPs detected and quantified in surface waters (excel sheet).

Table S10: Detected antibiotic TPs together with their estimated form as well as the concentration (excel sheet).

Table S11: Other antibacterial TPs detected in surface waters (excel sheet).

Table S12: Information about associated antiviral parent compounds (excel sheet).

Table S13: Comparison of RQ_{AMR}, $RQ_{species}$, mutagenicity, carcinogenicity, persistence, mobility (solubility and log K_{OC}) and log BCF between TPs and parent compounds (excel sheet).

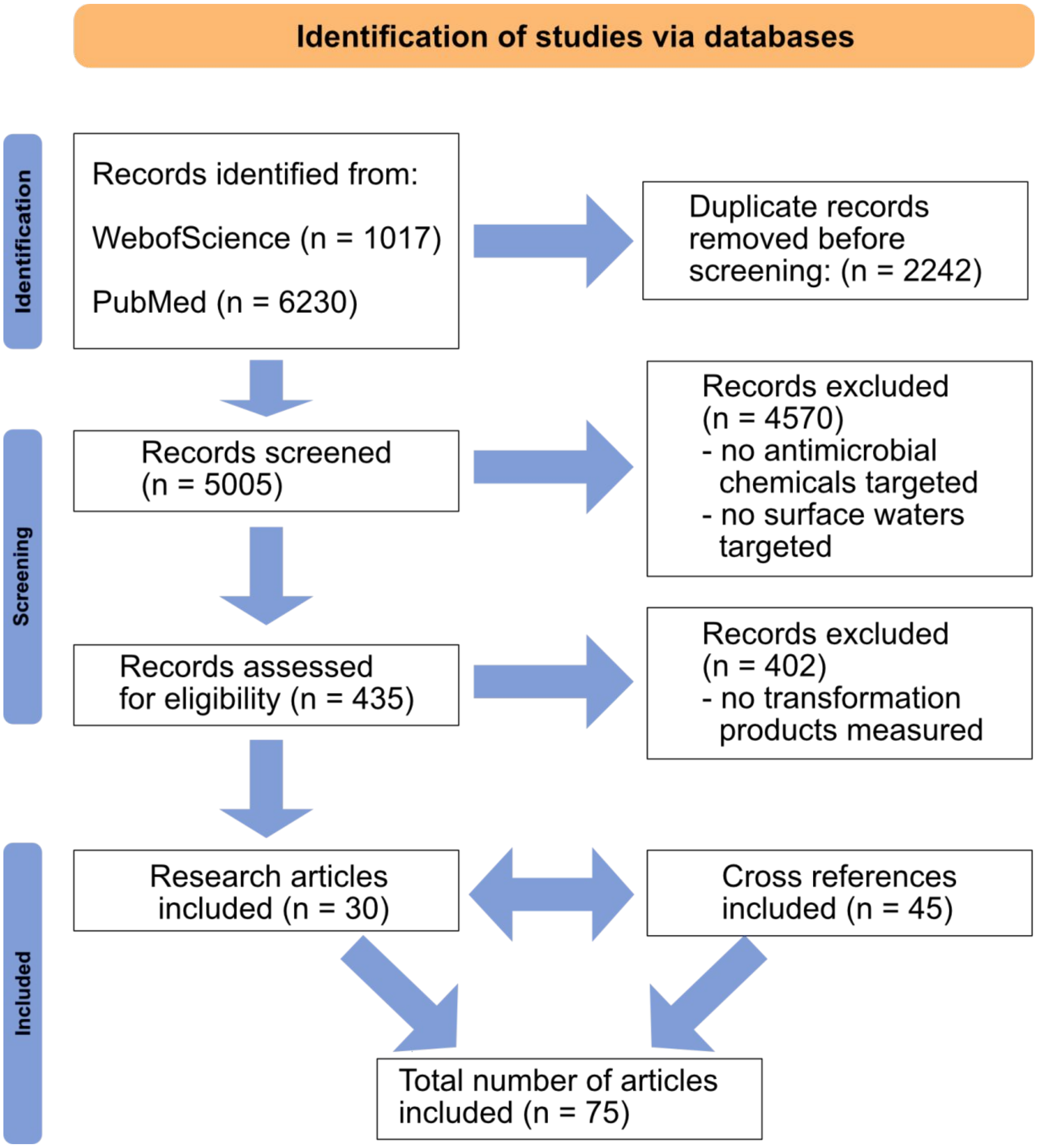

Figure S1: Systematic literature search flow diagram with inclusion and exclusion criteria.

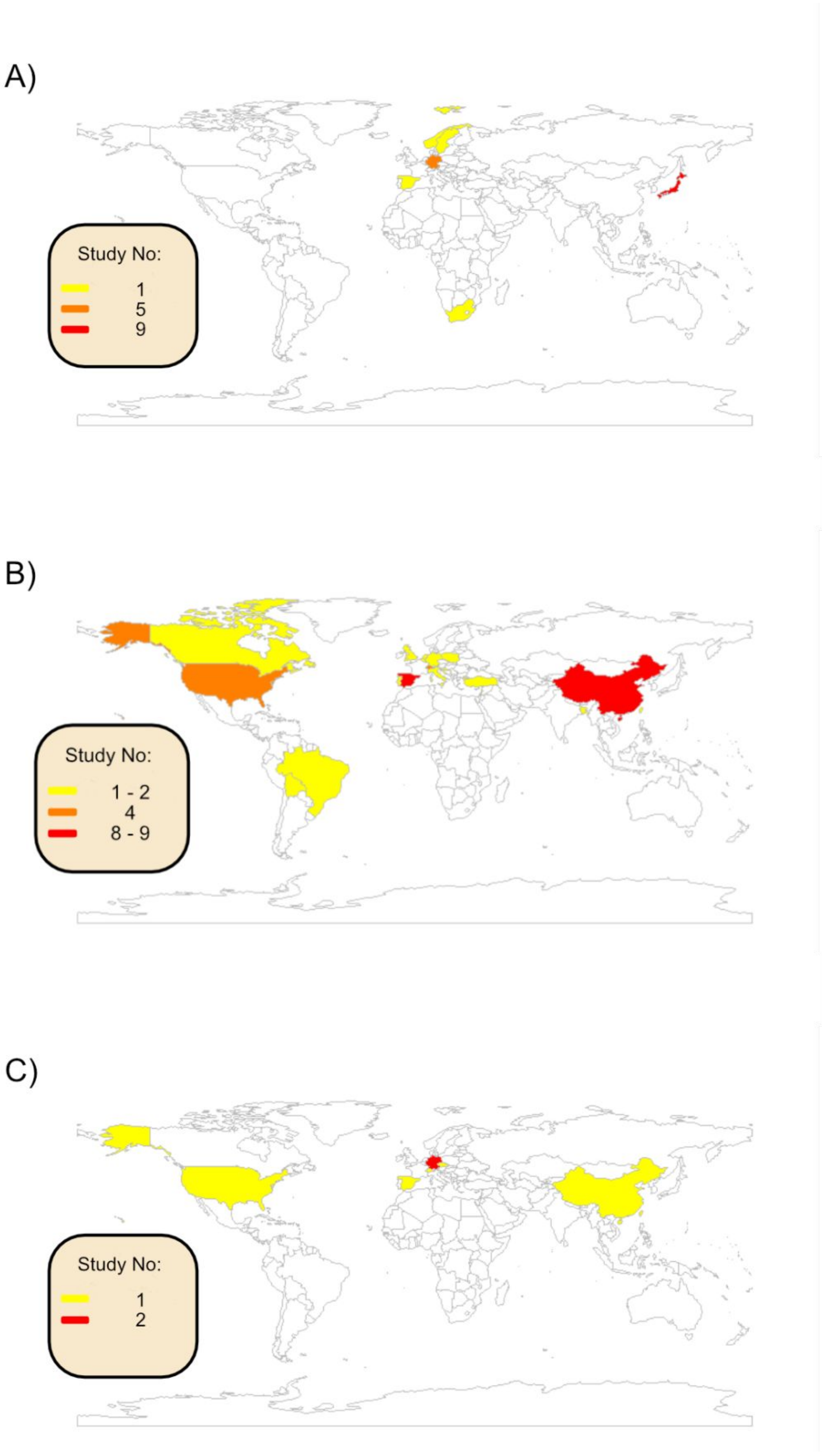

Figure S2: Number of studies that detected or quantified TPs in surface waters for antivirals (A), antibiotics (B), and other antibacterials (C).

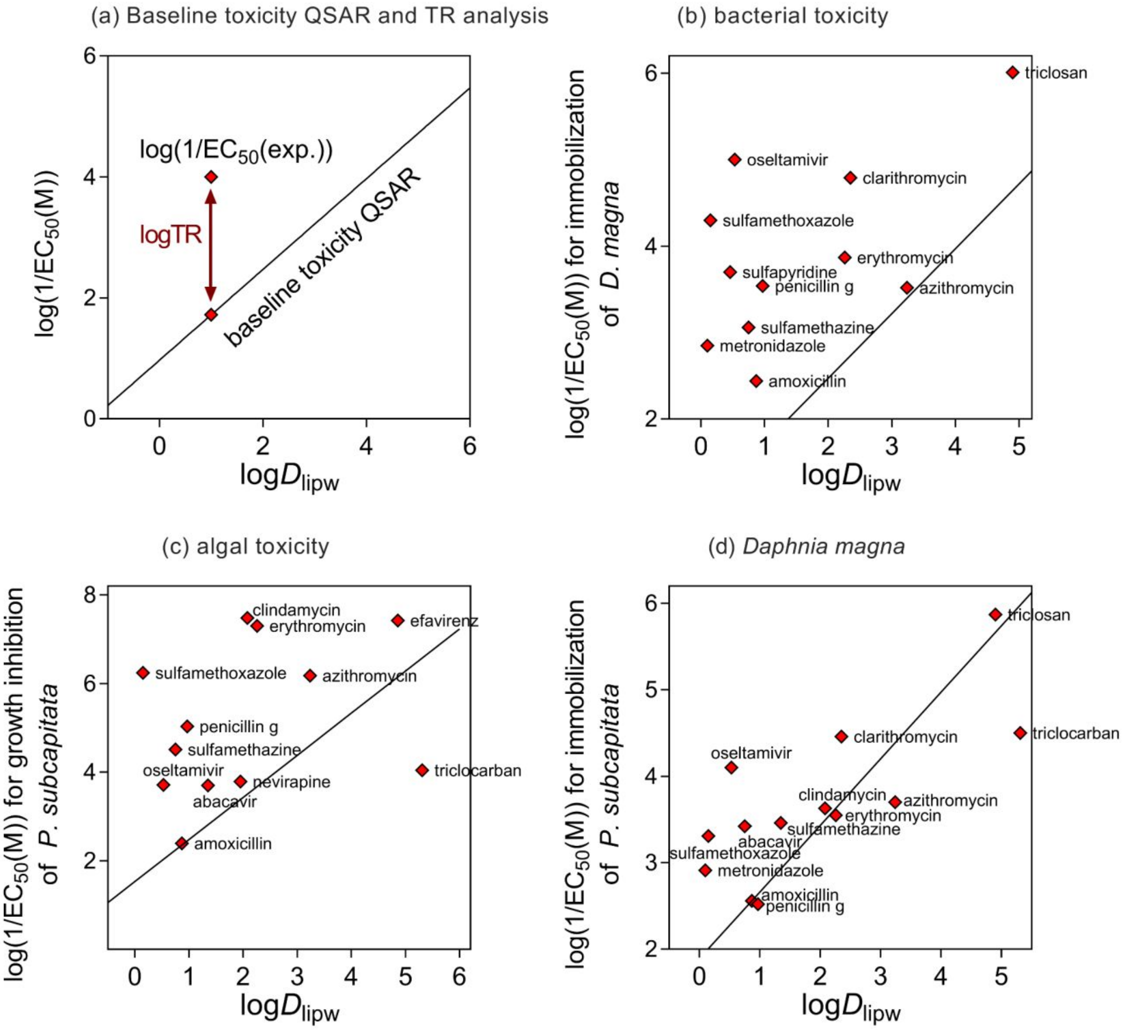

Figure S3: (a) Theoretical difference between predicted baseline toxicity and specific toxicity endpoint; parent antimicrobial toxicity towards (b) *A. fischeri*, (c) *P. subcapitata*, and (d) *D. magna* including baseline toxicity prediction (line) using Equations 3 to 5.

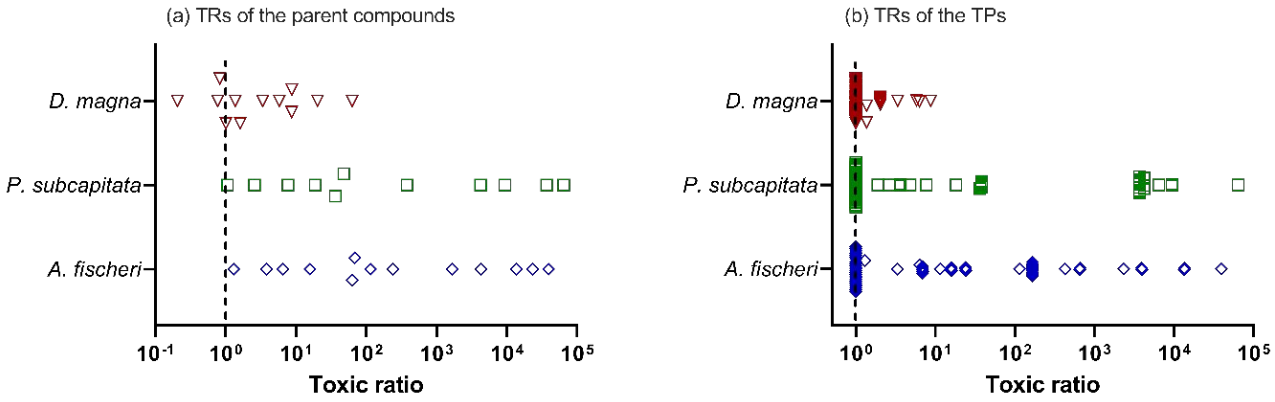

Figure S4: Range of toxic ratios TR of (a) the parent antibiotics and (b) the transformation products TP.

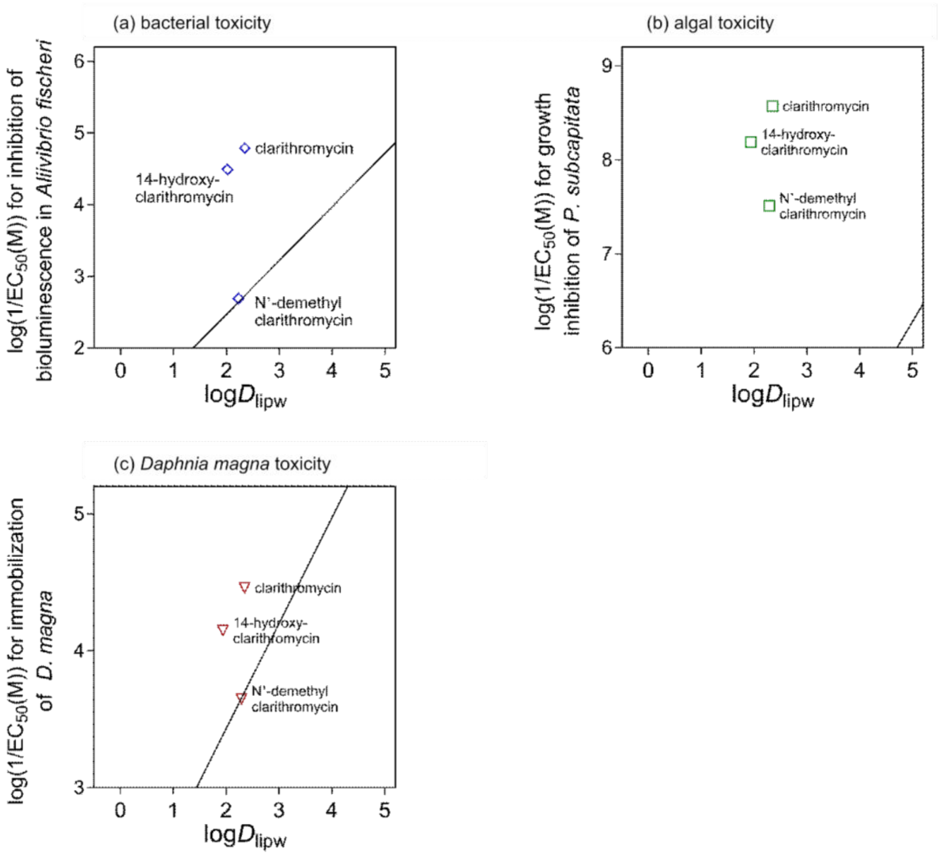

Figure S5: Estimated specific toxicity of clarithromycin and its TPs towards *A. fischeri* (a), *P. subcapitata* (b), *D. magna* (c).

Table S1: Summary of selected literature, identified transformation products (TPs) in surface waters, respective compound class, family and parent compound of the TPs, TP concentration range, instrumentation and country of the studied surface water.

Table S2: Similarity evaluation and RQ_{AMR} calculations.

Table S3: EC_{50}, PNEC and RQ estimations of the compiled TPs.

Table S4: Database information on mutagenicity and carcinogenicity to validate predictions.

Table S5: Mutagenicity and carcinogenicity literature of parent compounds to validate the prediction.

Table S6: Parameters assessing model performance of mutagenicity and carcinogenicity predictions.

Table S7: Mutagenicity and carcinogenicity estimations of compiled TPs and respective parent compounds.

Table S8: TPs with characterization parameters and scores (light grey filled) for prioritization.

Table S9: Antiviral TPs detected and quantified in surface waters.

Table S10: Detected antibiotic TPs together with their estimated form as well as the concentration.

Table S11: Other antibacterial TPs detected in surface waters.

Table S12: Information about associated antiviral parent compounds.

Table S13: Comparison of RQ_{AMR}, $RQ_{species}$, mutagenicity, carcinogenicity, persistence, mobility (solubility and log K_{OC}) and log BCF between TPs and parent compounds.
